# Helical mutations in type I collagen that affect the processing of the amino-propeptide result in an Osteogenesis Imperfecta/Ehlers-Danlos Syndrome overlap syndrome

**DOI:** 10.1186/1750-1172-8-78

**Published:** 2013-05-21

**Authors:** Fransiska Malfait, Sofie Symoens, Nathalie Goemans, Yolanda Gyftodimou, Eva Holmberg, Vanesa López-González, Geert Mortier, Sheela Nampoothiri, Michael Bjorn Petersen, Anne De Paepe

**Affiliations:** 1 Center for Medical Genetics, Ghent University Hospital, De Pintelaan 85, Ghent 9000, Belgium; 2 Child Neurology, University Hospitals Leuven, Leuven, Belgium; 3 Department of Genetics, Institute of Child Health, Athens, 11527, Greece; 4 Department of Clinical Genetics, Sahlgrenska University Hospital, Gothenburg, SE-413 45, Sweden; 5 Unidad de Genetica Medica, Servicio de Pediatria, Hospital Universitario Virgen de la Arrixaca, El Palmar, Murcia, Spain; 6 Department of Medical Genetics, Antwerp University Hospital, University of Antwerp, 2650 Edegem and Ghent University, Ghent, Belgium; 7 Department of Pediatric Genetics, Amrita Institute of Medical Sciences and Research Center, AIMS Ponekkara PO, Cochin, Kerala, India; 8 Department of Clinical Genetics, Aalborg University Hospital, Aalborg, 9000, Denmark

**Keywords:** Ehlers-Danlos syndrome, Osteogenesis Imperfecta, Type I collagen, Arterial fragility, Genotype, Phenotype

## Abstract

**Background:**

Whereas mutations affecting the helical domain of type I procollagen classically cause Osteogenesis Imperfecta (OI), helical mutations near the amino (N)-proteinase cleavage site have been suggested to result in a mixed OI/Ehlers-Danlos syndrome (EDS)-phenotype.

**Methods:**

We performed biochemical and molecular analysis of type I (pro-) collagen in a cohort of seven patients referred with a clinical diagnosis of EDS and showing only subtle signs of OI. Transmission electron microscopy of the dermis was available for one patient.

**Results:**

All of these patients harboured a *COL1A1* / *COL1A2* mutation residing within the most N-terminal part of the type I collagen helix. These mutations affect the rate of type I collagen N-propeptide cleavage and disturb normal collagen fibrillogenesis. Importantly, patients with this type of mutation do not show a typical OI phenotype but mainly present as EDS patients displaying severe joint hyperlaxity, soft and hyperextensible skin, abnormal wound healing, easy bruising, and sometimes signs of arterial fragility. In addition, they show subtle signs of OI including blue sclerae, relatively short stature and osteopenia or fractures.

**Conclusion:**

Recognition of this distinct phenotype is important for accurate genetic counselling, clinical management and surveillance, particularly in relation to the potential risk for vascular rupture associated with these mutations. Because these patients present clinical overlap with other EDS subtypes, biochemical collagen analysis is necessary to establish the correct diagnosis.

## Background

Type I collagen is the most abundant extracellular matrix (ECM) protein in humans and the major structural protein of bone, tendon, skin and cornea. It is a heterotrimer consisting of two α1-chains and one α2-chain, encoded by *COL1A1* (MIM: 120150) and *COL1A2* (MIM: 120160) respectively. The precursor form, type I procollagen, contains a central helical domain, flanked by an amino-(N) and carboxy-(C) terminal propeptide. The central helical domain consists of a repeating [Gly-X-Y] triplet in which glycine is the only amino acid small enough to reside within the sterically restricted inner aspect of the helix. Type I procollagen molecules are synthesized and folded in the rough endoplasmic reticulum, where they are modified by hydroxylating and glycosylating enzymes and are then secreted into the ECM. After cleavage of the N- and C-terminal propeptide by specific N- and C-proteinases, they are converted to mature collagen molecules, which self-assemble into fibrils.

Mutations in the genes encoding type I procollagen produce a range of disorders, which include autosomal dominant (AD) osteogenesis imperfecta (OI) and the rare arthrochalasis subtype of EDS. OI comprises a spectrum of mild to lethal phenotypes, characterized by a variable degree of bone fragility with susceptibility to bone fractures, growth deficiency, blue sclerae, hearing deficit and dentinogenesis imperfecta. Over 1000 distinct mutations in the primary genetic sequence of *COL1A1* and *COL1A2* have been identified in one or different OI variants [1,2] (Dalgleish R.: Osteogenesis Imperfecta Variant Database (https://oi.gene.le.ac.uk, accessed January 21^st^ 2013). The most frequent type of mutations are missense mutations that cause the substitution of one of the crucial helical glycine residues by a bulkier amino acid, nonsense mutations which result in a non-functional *COL1A1* allele or splicing mutations which lead to exon-skipping [3]. They are widespread over the *COL1A1* and *COL1A2* genes, including mainly the helical- and, to a lesser extent, the C-propeptide-encoding region of the genes.

In contrast, the rare EDS arthrochalasis subtype results from a specific type of mutation in either *COL1A1* or *COL1A2,* which causes complete or partial skipping of exon 6, encoding the procollagen type I-N-proteinase cleavage site [4]. Due to loss of the cleavage site, the processing of either the proα1(I) or the proα2(I) N-propeptide is abolished and incompletely processed procollagen chains, in which the C-propeptide but not the N-propeptide has been cleaved off, are secreted and incorporated into the growing collagen fibrils. The phenotype of this rare EDS variant is characterized by severe, generalized joint hypermobility and recurring joint dislocations, including bilateral congenital hip dislocation (CHD), skin hyperextensibility, atrophic scarring, mild dysmorphic features, short stature, blue sclerae and osteopenia [5,6].

Occasionally, patients are encountered who display a phenotype that combines clinical manifestations of both OI and EDS. The few published reports on such patients with a mixed OI/EDS phenotype demonstrate that most of them harbour a mutation in the most N-terminal part of the type I collagen helical region in either the α1- or α2- chain, which affects to some extent proper processing of the N-propeptide [7-12].

Here we substantially extend the patient cohort with an OI/EDS overlap syndrome and provide a comprehensive overview of the clinical, biochemical and molecular characteristics of type I collagen mutations, which cause this phenotype. Our data show that some patients harbouring a mutation within this region of the type I collagen molecule predominantly present with an EDS phenotype that resembles, but is distinct from other EDS subtypes, both in clinical spectrum and severity, as in the underlying collagen protein defect.

## Material and methods

### Clinical information

For all probands clinical information was obtained from physical examination by one of the authors. Informed consent from the probands and/or their legal guardians was obtained in accordance with requirements of the Local Ethics Committees. Skin biopsies were taken from the probands’ inner aspect of the upper arm for the establishment of a fibroblast culture. In proband P3, part of the skin biopsy was prepared for transmission electron microscopy (TEM).

### Biochemical studies and ultrastructural studies

Fibroblast cell culture, steady state collagen labelling and sodium dodecyl sulfate polyacrylamide gel electrophoresis (SDS-PAGE) analysis were performed as described previously [13]. Briefly, at confluency the patients’ fibroblasts were labelled with ^14^C-Proline [14] and intracellular collagen and secreted (pro)collagen proteins were separated on 6% SDS-PAGE gels. The SDS-PAGE-gels were processed for fluorography, dried, and exposed to an X-ray film.

In order to examine the conversion of procollagen to collagen by pericellular enzymes, pulse chase analysis was performed as previously described [7]. Processing of secreted procollagens to mature collagen by the procollagen-I-C-proteinase BMP-1 and the procollagen-I-N-proteinase ADAMTS2 was studied over a 5-day period. Dermal fibroblasts were labelled with ^14^C-proline for 20 hr and medium was harvested at 24-hr intervals. Medium procollagen samples were isolated, electrophoresed on 6% SDS-PAGE gels and visualized by autoradiography.

In proband P3 part of the skip biopsy was prepared for TEM following fixation in 5% glutaraldehyde in phosphate buffer at pH 7.4, then fixed in 1% osmium tetroxide for 60 min, dehydrated in acetone and embedded in Epoxy-resin. Semi-thin sections were stained with 2% methylene blue in 1% Borax. Examination was performed using a Zeiss Microscope.

### Mutation Identification

Total RNA was isolated from cultured skin fibroblasts from the probands by TRIZOL (Life Technologies). For the conversion to cDNA, moloney murine leukemia virus reverse transcriptase (M-MLV-RT) was used in combination with random hexanucleotide primers. For sequencing at the genomic DNA (gDNA) level, gDNA was extracted from cultured skin fibroblasts (DNeasy-Invitrogen) according to the manufacturer’s instructions. The coding regions (cDNA level) and exons and flanking introns (gDNA level) of *COL1A1* and *COL1A2* were PCR amplified and were separated on agarose (PCR primers available upon request). The amplimers were sequenced using the ABI3730XL sequencer.

Nucleotides were numbered starting from the first base of the initiation codon (ATG) of the cDNA reference sequence. Reference cDNA sequence is based on Genbank Accession no.: NM_000088.3 for *COL1A1* and NM_000089.3 for *COL1A2*. Amino acid residues were numbered from the first methionine residue of the reference sequence.

## Results

### Clinical Description and molecular findings

All probands were referred with a clinical diagnosis of Ehlers-Danlos syndrome and presented a phenotype predominated by generalized joint hyperlaxity and skin hyperextensibility and/or translucency and, in some of them, also signs of vascular fragility. In addition, all probands presented with relatively short stature, blue sclerae and mild signs of bone fragility with osteopenia and/or a history of infrequent fractures. These symptoms however did not predominate the clinical picture, and did not elicit a clinical diagnosis of OI.

**Figure 1:**
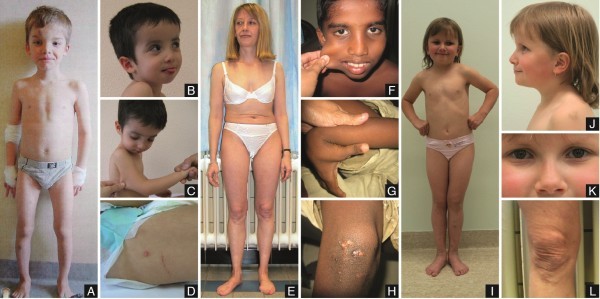
**Clinical features of the probands. A**: P1 at the age of 7 yrs. Note hypertelorism, several bruises on forehead and shins, mild pectus excavatum and slightly bowed legs. **B-D**: P2. Note the mildly blue sclerae, prominent forehead, mildly hypertrophic scarring and skin hyperextensibility. **E&L**: P3 at the age of 37 yrs. Note the short trunk and mildly atrophic scars on the knees. **F-H**: P5 at the age of 10 yrs: skin hyperextensibility, generalized joint hyperlaxity and mildly hypertrophic cicatrisation. **I-K**: P7 at the age of 7 yrs. She displays blue sclerae and translucent skin. Note the multiple ecchymoses and hematomas on the legs.

Available clinical findings of the probands are summarized in Table 1 and illustrated in Figure 1.

**Table 1:** Clinical characteristics of the probands

	**P1**	**P2**	**P3**	**P4**	**P5**	**P6**	**P7**
**Patient identifier OI variant database**	**AN_001001**	**AN_001954**	**AN_001096**	**AN_001060**	**AN_001955**	**AN_001062**	**AN_001956**
Gene	*COL1A1*	*COL1A1*	*COL1A2*	*COL1A2*	*COL1A2*	*COL1A2*	*COL1A2*
Exon	7	8	7	8	9	12	14
Mutation	p.(G188D)	p.(G203C)	Skip ex 7	p.(G109D)	Skip ex 9	p.(G196V)	Skip ex 14
Sex	M	M	F	M	M	F	F
Stature	<3rd centile	3rd centile	<3rd centile	NA	<3rd centile	3rd centile	3rd-10th centile
**Skin/soft tissue**							
Hyperextensibility and/or translucency	+	+	+	+	+	+	+
Delayed wound healing, abnormal scarring	+	+	+	-	+	-	-
Easy bruising	+	+	+	+	+	+	+
**Muscle and joints**							
Hypotonia	+	+	+	+	-	+	-
Hypermobility	+	+	+	+	+	+	+
Dislocations	+	+	+	-	+	-	-
Scoliosis	-	+	-	-	-	-	-
CHD	-	-	-	-	-	-	-
**Bone**							
Fractures	+ pelvis/vertebrae	+ (3x)	+ (late-onset)	-	+ (2x)	+ (4x)	+2x greenstickfracture
**Cardiovascular**							
Echocardiography	ASD	Aortic dilation	NL	NL	NL	NL	ND
Arterial rupture	+	-	-	+	-	-	-
**Other**							
Blue sclerae	+	+	+	+	+	+	+
Hernia	-	Inguinal	-	-	-	-	-

ASD: atrial septum defect.

CHD: congenital hip dislocation.

NL: normal.

ND: Not done.

Proband P1 (Figure 1A) was born at term after an uneventful pregnancy and delivery. In early childhood he displayed generalized joint hypermobility, low muscle mass and a mild delay in neuromotor development. He bruised easily and at age 5 yrs, he suffered an epidural hematoma with intraspinal hemorrhage following a relatively mild trauma, which raised suspicion of vascular EDS. Clinical investigation at age 6 yrs revealed a short-statured boy with pale blue sclerae, hypertelorism, a pale soft and hyperextensible skin with some atrophic scars and generalized joint hypermobility (Beighton score 8/9). He suffered one fracture of the foot and recurrent shoulder dislocations. Skeletal X-rays were normal, but bone densitometry at the age of 6 yrs showed marked osteoporosis of the vertebrae, with a DEXA Z-score of -3.4 SD. At the age of 12 yrs he had a fracture of the pelvis and compression of several vertebrae for which bisphosphonate treatment was started. Ultrasonography of the heart showed an atrial septum defect (ASD) with right ventricular dilatation. The ASD was surgically closed, however the first patch loosened, and the boy had to be re-operated.

Proband P2 (Figure 1B-D) was born at term after an uneventful pregnancy and delivery. His gross motor development was delayed due to muscle hypotonia. At age 2 yrs he developed a kyphoscoliosis, which progressed in the following years. He sustained three fractures, of the right elbow, and of both femurs, before the age of 5 yrs, for which treatment with biphosphonates was started. X-rays showed osteopenia. At the of age 5 yrs 10 months, he displayed hyperextensible skin, abnormal wound healing, easy bruising, hyperextensible joints (Beighton score 9/9), pectus excavatum, long fingers and toes and inguinal hernia. In addition he displayed blue sclerae, macrocephaly, and short stature. Based on these findings he was clinically suspected to have EDS kyphoscoliotic type. At age 8 yrs, dilation of the aorta (+2.02 Z-score) was noted.

Proband P3 (Figure 1E&L) was born at term in breech position and presented with a shoulder dislocation. During childhood marked joint hypermobility, repetitive ankle distortions and easy bruising were noted. Later on, in early adulthood, she suffered from recurring dislocations of the wrists, knees and ankles and had a few late-onset fractures (toe, coccyx and vertebral compression fracture at adult age). Clinical investigation at age 37 yrs showed a short-statured woman with pale blue sclerae, a soft and mildly hyperextensible skin and a small, distended post-operative scar on the right knee. Joint hypermobility was generalized with a Beighton score of 8/9. Ultrasonography of the heart revealed mild mitral valve insufficiency. Bone densitometry at the age of 36 yrs showed marked osteoporosis of the vertebrae, with a DEXA Z-score of -3.5 SD. The probands father and paternal grandmother both presented short stature, blue sclerae, easy bruising and joint dislocations.

**Figure 2:**
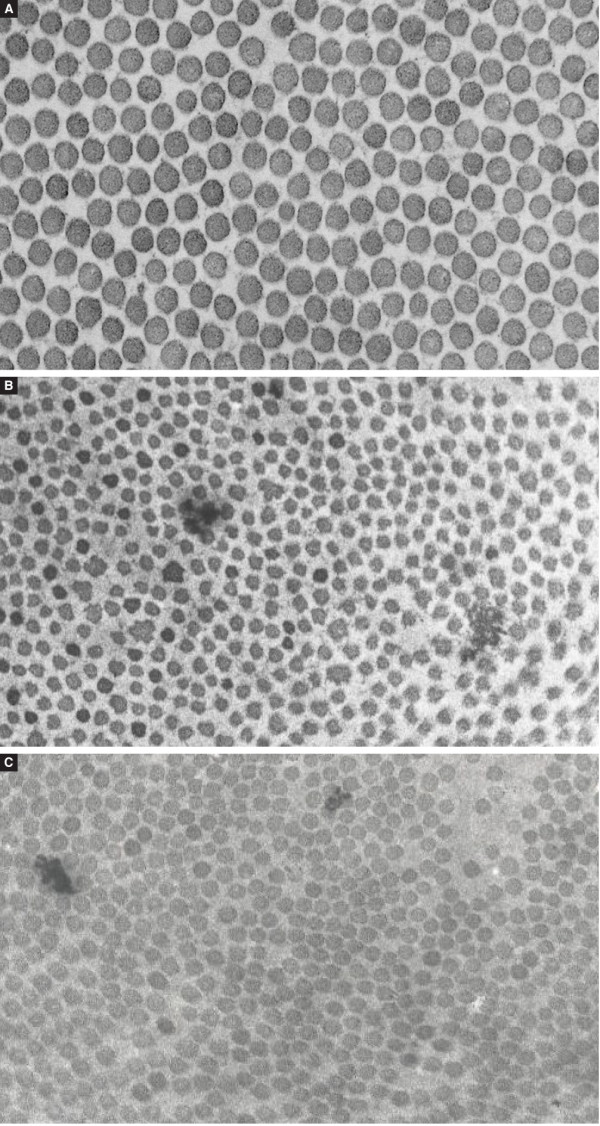
**Transmission electron microscopy of collagen dermal fibrils. A**: Normal control. Fibril diameter is 97,1 +/- 8,1 nm (original magnification 45.000x). **B**: EDS arthrochalasis subtype (VIIB): complete retention of the N-propeptide of the α2(I)-procollagen chain is caused by skipping of the N-proteinase cleavage site itself, resulting in incorporation of pN-collagen molecules into the collagen fibrils in the extracellular matrix. This causes loss of the normal collagen fibril morphology, with decreased fibril density, decreased fibril diameter (43.82 +/- 6.09 nm), irregular contours and occasional cauliflowers (original magnification 45.000x). **C**: OI/EDS-proband P3: The N-proteinase cleavage site remains intact, but processing of the type I procollagen N-propeptide is delayed, resulting in abnormal collagen fibrils, with irregular borders and diameters (73.01 +/- 5.88 nm) that are smaller than those from controls but larger than for patients with EDS arthrochalasis type. Fibril contours are only slightly irregular and fibril density is decreased (original magnification 45.000×).

Transmission electron microscopy was performed at age 37 yrs on a skin biopsy and showed lower collagen fibril density in the dermis than in a skin biopsy from a control subject. Collagen fibrils had a circular cross-section, but some fibrils displayed a slightly irregular contour. No cauliflower deformations were observed. The fibril diameter was distinctly smaller (73.01 +/- 5.88 nm) than in that of the control sample(s) (97.10 +/- 8.1 nm) (Figure 2) (courtesy of Prof Dr Yves Vanderhaeghen, Department of Dermatology, Ghent University Hospital, Belgium).

Proband P4 was born at term by ceasarian section. He presented dark blue sclerae, marked generalized joint hypermobility, and a hyperextensible, thin and transparent skin with visible veins on the thorax. At birth his height was at the 75^th^ centile. On the 3^rd^ day of life he developed tonic-clonic seizures with opisthotonus. Ultrasonography of the brain revealed a massive intracranial bleeding, reason for which a diagnosis of vascular EDS was suspected. At 18 months extremely lax joints, pes planovalgus and mildly hyperelastic, soft skin were noticed. He also presented long and thin fingers. At age 7 yrs he had secondary hydrocephalus, treated with a shunt, axial hypotonia, mild spastic right hemiplegia, focal seizures and psychomotor delay. Ultrasonography of the heart was normal and skull X-ray was negative for wormian bones. At the age of 6 yrs 3 months he exhibited severe lumbar osteopenia with Z-score -2,1 SD and incipient generalized osteopenia of -1,1 SD. At 7 yrs weight was at the 30th centile, length at the 5^th^ centile, and head circumference at the 3rd centile. While his skin was no longer hyperelastic, there was some bruisability, slow wound healing and he presented one atrophic scar on his arm. His joints were still very lax.

The proband’s mother displayed pale blue sclerae, thin face with thin and pinched nose, wrinkly skin and a papyraceous scar on the elbow, extensive striae gravidarum and arachnodactyly. At 18 months of age she suffered a shoulder dislocation. At adult age, she still presented extreme generalized joint laxity. She had mitral valve prolaps with myxomatous degeneration and mild regurgitation. DEXA bone densitometry revealed femoral osteopenia with Z-score -1,55 SD while the whole body Z-score was normal. The maternal grandmother of the proband died at the age of 38 yrs due to rupture of an ascending aorta aneurysm. She also had generalized joint hyperlaxity, short stature and a thin, fragile skin, as did her brother.

Proband P5 (Figure 1F-H) was born after an uneventful pregnancy and delivery. At birth, joint hyperlaxity with a dislocatable patella was noted. At age 9 yrs 5 months he presented a soft, doughy and hyperextensible skin, with increased palmar wrinkling, and mild hypertrophic scarring. He displayed generalized joint hyperlaxity with a Beighton score of 9/9, and a history of dislocated thumbs and temporomandibular joints. He had a slightly blue scleral hue, rather short stature, and sustained a supracondylar humeral fracture at age 2.5 yrs, with a second fractures at the same site 6 months later.

Proband P6 was born at term with a height of 47 cm (<3rd centile) and presented with easy bruising generalized joint hypermobility and muscle hypotonia with delay in gross motor development, reason for which she was referred to a neurologist. She suffered three nose fractures and sustained a fracture of the foot after mild trauma. Clinical examination at age 4 yrs showed a short-statured girl with blue sclerae and a pale round face. She displayed a hyperextensible, thin and translucent skin with visible veins on the thorax, as well as marked generalized joint hypermobility (Beighton score 9/9). She bruised easily and displayed multiple small hematoma’s on the legs upon clinical examination. Skeletal X-rays revealed osteopenia of the spine and mild bowing of the tibia and fibula. Ultrasonography of the heart was normal. Family history was negative for connective tissue weakness.

Proband P7 (Figure 1I-K) was born after an uneventful pregnancy and delivery. She sustained two greenstick fractures of the ankles at age 4 and 6 yrs respectively. At age 6 yrs, she was clinically suspected to have vascular EDS, because of a thin, translucent skin with a somewhat acrogeric aspect on hands and feet and marked bruising after minor trauma. A small atrophic scar on the leg and hyperlaxity of the finger joints was noted on clinical examination. She was rather short-statured (height between 3^rd^ and 10^th^ centiles) with deep blue sclerae, macrocephaly head circumference at 97^th^ centile), frontal bossing, lack of peri-orbital fat, and pes planus. A skeletal survey at age 8 yrs 4 months revealed Wormian bones in the skull, generalized osteopenia and mild bowing of the tibia and fibula diaphyses. Family history was unremarkable for connective tissue disorders.

### Steady-state biochemichal findings

**Figure 3:**
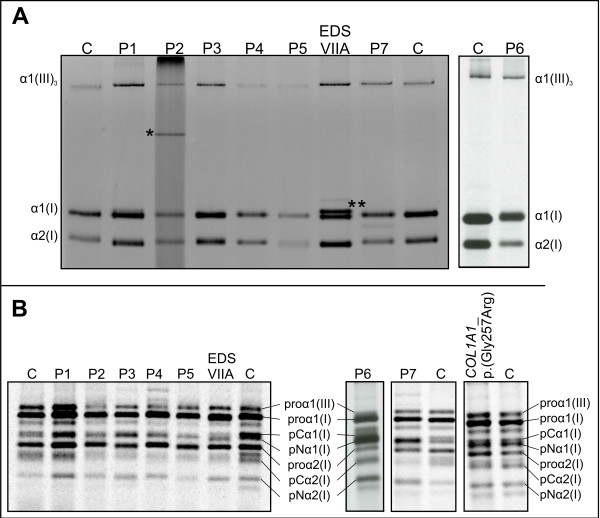
**SDS-PAGE analysis of secreted **^**14**^**C-labeled (pro)collagen molecules from dermal fibroblasts. **Secreted procollagen molecules were extracted from the medium fraction of the cell cultures. The globular N- and C-propeptide of the procollagen molecules were removed by pepsin-digestion, resulting in mature triple helical collagen proteins. Lane numbers correspond to the patient’s numbers in the text; “C” corresponds to control samples. panel **A**: medium fraction. P2: note the extra band of higher molecular weight, suggestive for presence of mutant α1(I)-collagen dimers ( indicated by *). EDS VIIA: patient with EDS arthrochalasis type, due to a *COL1A1* exon 6 skip: note the “doublet” for the α1(I) collagen chain that corresponds to mature α-chains and the incompletely cleaved pNα1(I)-chains (indicated by **) respectively. This doublet is absent in the OI/EDS patients. panel **B**: medium procollagen fraction. Compared to the control sample, the intensity of the bands representing the pNα1(I) chain is increased, whereas the intensity of the bands representing the pCα1(I) and the mature α1(I)-chain is decreased. This is caused by a disturbed balance in the normal kinetics of the delayed N-processing and the unhampered C-processing of type I procollagen. This pattern is similar to what is observed in the patient with EDS arthrochalasis type (EDS VIIA). In the right panel, the medium procollagen fraction is shown from a patient with classic OI, without EDS-signs, with p.(Gly257Arg) substitution in exon 11 of *COL1A1*. Here, intensity of the bands representing the pNα1-, pCα1- and mature α1 chains of type I collagens is comparable to that of the control.

SDS-PAGE analysis of collagens type I and III in the medium and the cell layer fraction showed a normal aspect of the band representing type III collagen in all probands, making a diagnosis of vascular EDS highly unlikely (Figure 3A). In contrast, the pattern of pepsin-digested type I collagen retained in the cells, and secreted in the medium was abnormal for probands P1, P2, P5 and P6. Slight differences in the electrophoretic migration pattern were visible among the different patient samples. In P1 the bands representing the mature α-chains of type I collagen were significantly broadened in the cell layer (not shown) and in the medium fraction, and migrated somewhat faster in comparison to the control. In P2, an extra band of higher molecular weight, suggestive for presence of mutant α1(I)-collagen dimers, was observed in both cell layer (not shown) and medium fraction. In P5 and P6 a fuzzy and slightly broadened aspect of the bands representing the α2(I)-collagen chains (in P5) or both α(I) collagen chains (in P6) was observed in both cell layer (not shown) and medium.

SDS-PAGE analysis of procollagen type I and III, extracted from medium and cell layer of the skin fibroblast culture, showed a normal aspect of the band representing type III procollagen in all probands. However, in all probands, an increased intensity of the bands representing the pNα1 chains of type I procollagen was observed compared to the control sample, whereas a decreased intensity of the bands representing the pCα1(I) and the mature α1(I) collagen chains was observed compared to the control sample, which suggested that the processing of the N-propeptide was delayed (Figure 3B). In contrast, no differences in intensity of the bands representing the pNα1- and pCα1-chains were observed in a patient with classic OI, without overt EDS-signs, with a p.(Gly257Arg) substitution in exon 11 of *COL1A1*.

### Pulse chase analysis

**Figure 4:**
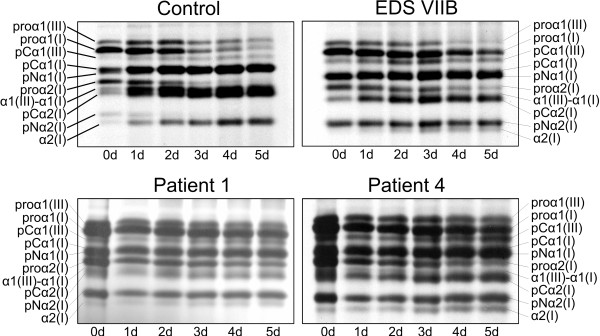
**Pericellular processing of procollagen. **The conversion of a pulse of [^3^H]-procollagen to collagen was followed over 5 days (d0-d5). The pulse chase analyses are shown for a control sample, P1, P4, and a patient with EDS arthrochalasis type due to an exon 6 skip in *COL1A2 *(EDS VIIB). In the control sample a clear decrease of the bands representing the pNα2(I) chain and increase of bands representing the mature α2(I) chains is observed over 5 days. Processing of the N-propeptide of type I collagen is delayed in both P1, P4 and in the patient with EDS VIIB. This is best seen by the accumulation of the bands representing pNα2(I) chains and a slower increase of mature α2(I) chains by day 5, compared to control.

In order to evaluate the conversion of procollagen to collagen, a pulse chase analysis was performed. The rate of conversion of procollagen to collagen by the C-proteinase (BMP-1) and the N-proteinase (ADAMTS2) processing enzymes in cell culture was examined over a 5-day period in P1, P3, P4 and P6 and in control samples. Processing of pN(I)-collagen to mature collagen was delayed in comparison to the control sample. This was most clearly visible for the conversion of pNα2(I)- to α2(I)-collagen. In contrast, the processing of the C-propeptide occurred at normal rate (Figure 4).

### Molecular findings

**Figure 5:**
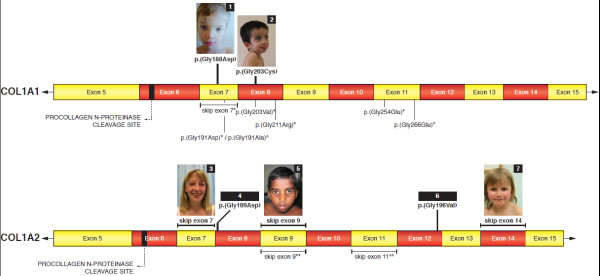
**Schematic localisation of the OI/EDS-associated *COL1A1 *and *COL1A2 *mutations reported in the present study (on top of bar) and previously published (below bar).** * [7], ° [14], ** [10,11], °° [8].

In view of the abnormalities in electrophoretic migration pattern of type I (pro)-collagen, sequencing of *COL1A1* and *COL1A2* was performed in all probands. These analyses allowed identification of heterozygous mutations in the triple helical region close to the procollagen type I N-proteinase cleavage site in all probands (Table 1, Figure 5).

In P1 and P2 a *COL1A1* missense mutation resulting in a glycine substitution was identified, in respectively exon 7 (c.563G>A; p.(Gly188Asp)) for P1 and in exon 8 (c. c.607G>T; p.(Gly203Cys)) for P2.

In P4 and P6 a *COL1A2* missense mutation resulting in a glycine substitution was identified, in respectively exon 8 (c.326G>A; p.(Gly109Asp)) for P4 and exon 12 (c. 587G>T; p.(Gly196Val)) for P6.

In P3, P5 and P7 a *COL1A2* mutation resulting in a single in-frame exon skip was identified, including an exon skip (c.324+4delA) in P3, an exon 9 skip (c.432+4_432+7delAGTA) in P5 and an exon 14 skip (c.693+5G>A) in P7.

## Discussion

In this study we report on a cohort of patients with an OI/EDS overlap syndrome resulting from a mutation in the N-terminal part of the type I collagen helical domain. We show that, in contrast to what is usually seen for type I collagen mutations, these patients clinically present as EDS patients. Indeed, all patients described here share generalized joint hypermobility and dislocations, skin hyperextensibility and/or translucency, easy bruising and (mild) abnormal scarring as the predominant clinical features, although invariably associated with mild signs of OI, including short stature, blue sclerae and osteopenia or infrequent fractures. Nevertheless, none of these patients were clinically diagnosed with OI, suggesting that the features resulting from bone fragility were of lesser importance than the features resulting from the soft connective tissue weakness that is characteristic for EDS. Clinical overlap with other EDS subtypes, including the classic, hypermobility, vascular, arthrochalasis and kyphoscoliosis type, can hamper the correct diagnosis. Therefore, biochemical collagen studies, revealing a processing abnormality of type I (pro-) collagen, are particularly helpful in the diagnostic evaluation.

Only one study so far exists on patients with an OI/EDS overlap phenotype, caused by a mutation located within the 85 most N-terminal amino acids of the α1(I)-collagen helical region. Six patients with a glycine substitution and one patient with an exon 7 skip displayed a phenotype predominantly characterized by severe bone fragility, clinically diagnosed as OI type III or IV, in conjunction with generalized joint hyperlaxity and early progressive scoliosis, which was attributed to severe paraspinal laxity [7]. A small number of patients with a mixed OI/EDS phenotype has been reported with an exon-skip [8,10-12] or multi-exon-duplication [9] in the corresponding helical region of the α2(I)-collagen chain. These mutations cause a register shift of the mutant chain with respect to the normal chains. It was previously suggested, based on these published data, that the α1(I)-mutations in this region cause severe OI with joint hyperlaxity, whereas the α2(I)-mutations in the corresponding region result in an EDS arthrochalasis phenotype [7]. The data from the present study suggest that this distinction between the α1- and α2-collagen chain is not so strict, since, on the one hand, our patients with a *COL1A1* mutation display a milder, more EDS-like phenotype than the patient cohort reported by Cabral *et al.*[7] and, on the other hand, no significant difference in aspect or severity of the OI/EDS phenotype is observed between the patients with an α1(I)- versus an α2(I)-chain mutation. It can thus not be predicted from the phenotype whether the mutation resides in *COL1A1* or *COL1A2.* Our data also show that, besides exon skipping mutations, also glycine substitutions in this region of the α2(I)-collagen chain can be associated with an OI/EDS overlap phenotype. Our findings are in accordance with the studies from Cabral [7] and Makareeva *et al.*[13] who showed that mutations located within the 85 most N-terminal amino acids of the α1(I)- helical domain, which acts as a stabilizing “N-anchor” for collagen folding, result in unfolding of the helix and a conformational change of the adjacent N-propeptide cleavage site, slowing down the N-propeptide processing by procollagen I N-proteinase. For all mutations reported here, steady-state SDS-PAGE of type I procollagen showed abnormal processing of the procollagen I N-propeptide, a finding that was corroborated by the pulse chase assays. This delay in N-propeptide processing disturbs normal collagen fibrillogenesis, resulting in collagen fibrils with smaller diameters and irregular contour, as illustrated in proband P3. The EDS symptoms in the OI/EDS patients are attributed to the incomplete or delayed N-propeptide cleavage and incorporation of the resulting pN-collagen into matrix fibrils [7]. This hypothesis is corroborated by the observation that patients presenting with an OI phenotype but no overt features of EDS, who harbour a mutation within the same region of the α1- or α2-chain of type I collagen, do not show delayed type I procollagen N-propeptide processing on SDS-PAGE analysis.

It is noteworthy that several patients in this cohort display signs of vascular fragility, raising a suspicion of vascular EDS. Interestingly, intracerebral hemorrhage was reported previously in a OI/EDS patient with an α2(I)-exon 9 skip [11] and a patient with an α1(I)-p.(Gly191Ala) substitution was reported to suffer from multivessel cervical aneurysms [14]. Whether arterial fragility is more frequent in patients with a helical mutation located near the N-proteinase cleavage site remains to be determined, since aortic root dilatation, arterial dissection, and coronary and cerebral artery aneurysms are occasionally encountered in patients with mutations in other parts of the *COL1A1* or *COL1A2* gene, although, in general, they are infrequent [15-17]. Clinical follow-up studies will be needed to further delineate the natural history of the phenotype associated these OI/EDS mutations, but awareness for the development of vascular aneurysm or rupture in childhood and young adulthood is warranted. In the mean time surgical interventions and other invasive procedures should be performed with the utmost care and in consideration of increased risk for vascular fragility.

## Conclusion

In conclusion, we demonstrate that *COL1A1* and *COL1A2* single exon skips and missense mutations, resulting in glycine substitutions, which are located in the type I collagen helical domain near the N-propeptide cleavage site, affect proper N-propeptide processing and result in a mixed OI/EDS phenotype. Clinically these patients present EDS features that overlap with many other EDS subtypes. OI features on the other hand may be very mild, and as a result involvement of type I collagen is not always suspected. Biochemical collagen analysis is a powerful tool in the diagnostic work-up of these conditions as it helps in the differential diagnosis with other EDS subtypes. Furthermore it may point towards type I procollagen processing defects. Recognition of this distinct phenotype and confirmation of the underlying type I collagen defect are important for accurate genetic counselling, clinical management and surveillance, particularly in relation to the risk for vascular fragility.

## Consent

Written informed consent was obtained from the patient for publication of this report and any accompanying images.

## Competing interests

The authors declare no competing interests.

## Authors' contributions

FM, NG, GG, EH, VLG, GM, SN and MBP were responsible for clinical assessment of the patients. FM and ADP were instrumental in the experimental design and interpretation of the data. SS performed biochemical analyses and carried out DNA sequencing and analysis, receiving guidance and supervision from FM and ADP. FM drafted the manuscript. ADP revised and gave final approval for the manuscript to be published. All authors have read and approved the final manuscript.
